# A *Bacillus subtilis* strain with efficient algaecide of *Microcystis aeruginosa* and degradation of microcystins

**DOI:** 10.3389/fmicb.2024.1430097

**Published:** 2024-11-29

**Authors:** Yuanyuan Chen, Fei Xiong, Ying Zhu, Dongdong Zhai, Hongyan Liu, Lin Zhang, Ming Xia

**Affiliations:** ^1^Hubei Engineering Research Center for Protection and Utilization of Special Biological Resources in the Hanjiang River Basin, College of Life Sciences, Jianghan University, Wuhan, China; ^2^Yangtze River Fisheries Research Institute Chinese Academy of Fishery Sciences, Wuhan, China

**Keywords:** *Bacillus subtilis* S4, algicidal bacterium, algicidal properties, microcystins, *M. aeruginosa*

## Abstract

Global concerns over harmful cyanobacterial blooms brought on by eutrophication are now widespread. Aquatic ecological restoration techniques that use algicidal bacteria to control toxic algae show promise. A *Bacillus subtilis* S4 (S4) strain with strong *Microcystis aeruginosa* algicidal activity and the capacity to degrade microcystins (MCs) were successfully isolated and evaluated in this study. The dynamics of internal and extracellular MC concentration as well as the physiological response and morphological properties of *M. aeruginosa* were investigated in the *M. aeruginosa*/bacteria co-culture system. The findings demonstrated that when S4 density grew from 1 × 10^6^ cells/ml to 1 × 10^8^ cells/ml, the release of *M. aeruginosa* lysis and MCs was boosted; however, MCs dropped by approximately 90% within 18 h, regardless of bacterial density. Comparing the bacterial cell incubation system to the control and bacterial cell-free filtrate systems, the assessment of extracellular and intracellular MCs revealed a 95% reduction in MCs. The findings showed that 89% of MCs were decreased by bacterial cells, while 98% of *M. aeruginosa* cells were algaecided by bacterial metabolites. Sustainable eradication of *M. aeruginosa* and MCs has been accomplished by the combined efforts of the S4 strain and its metabolites. By secreting algicidal chemicals that are resistant to proteases, acid, base, and heat, the S4 strain indirectly acts as an algaecide. The S4 strain possesses a strong ability to break down MCs and a very effective and stable algaecide function, indicating that it can potentially treat eutrophic water with hazardous algae.

## 1 Introduction

Cyanobacteria blooms caused by eutrophication have emerged worldwide, leading to a series of issues, such as odor and microcystin (MC) pollution, which severely damage ecosystems and pose a threat to human health (Namsaraev et al., 2020; Zhang et al., 2023). Despite the growing body of research on cyanobacteria blooms, the mechanisms underlying their control and the mitigation of their toxins remain poorly understood. Hence, there is an increasing interest in cost-effective and environmentally friendly remediation technologies to control these blooms. However, these methods often fall short due to their non-selective nature, the potential for generating harmful by-products, and the risk of ecological imbalance (Song et al., 2023). As an effective method for eliminating cyanobacterial blooms, biological methods have received widespread attention due to their environmental friendliness, especially microbial-based methods (Pal et al., 2020). Among these, *Bacillus* species have emerged as promising candidates due to their broad-spectrum activity and minimal environmental impact.

Targeting cyanobacteria that cause blooms, approximately 151 cyanobactericidal bacterial strains have been reported, hailing from over 4 phyla, 12 classes, 26 orders, 35 families, and 49 genera (Yang C. et al., 2020). Predominantly, they belong to Cytophaga/Flavobacterium/Bacteroidetes or Gammaproteobacteria, such as *Alteromonas*, *Bacillus*, *Flavobacterium*, *Pseudoalteromonas*, *Pseudomonas*, and *Vibrio* (Wang et al., 2020). *Bacilli* emerge as the dominant group, constituting 31.1% of these strains (Yang C. et al., 2020). The majority of *Bacilli* are isolated from waters containing cyanobacteria, particularly from bloom-affected areas such as Taihu and Dianchi lakes (Liu et al., 2019; Tian et al., 2012). This supports the notion that algicidal bacteria may play a role in naturally regulating algal blooms, although the patterns and mechanisms remain largely unknown (Meyer et al., 2017). The target cyanobacteria for *Bacilli* are listed in Supplementary Table S1, with the vast majority (92.31%) capable of killing *Microcystis*, likely due to their common association with bloom formation and widespread use as target algae for screening cyanobactericidal bacteria. For *Bacilli*, a variety of prey spectra at different levels (e.g., species, genus) have been reported, with over half of the bacteria (84.62%) targeting only one species of algae, as shown in Supplementary Table S1. However, this also reveals a critical gap in our knowledge regarding the degradation of cyanotoxins, such as MCs, by these strains. While some strains show promise in algicidal activity, the majority of them lack specific details on MC degradation, highlighting a significant research need. The absence of comprehensive data on MC degradation is a notable shortfall, as the release of these toxins during algal blooms poses substantial ecological and health risks.

The application of algicidal bacteria, like other cell lysis methods, carries the potential risk of releasing harmful secondary metabolites, highlighting the necessity for strains capable of degrading MCs in addition to their algicidal effects (Massey and Yang, 2020). MCs are a type of stable cyclic heptapeptide that can inhibit the activity of liver protein phosphatase and induce liver cancer (Schreidah et al., 2020). These toxins can accumulate in aquatic food chains, posing a significant risk to both wildlife and human health due to their potent hepatotoxic effects. In addition to toxicity and stability, the overproduction of MCs is also a common concern, which is induced by environmental changes, such as light intensity, carbon and nitrogen levels, trace elements, and environmental toxicants (Brêda-Alves et al., 2021). These MC properties have attracted attention to various algaecide treatments. The persistence and toxicity of MCs have prompted the development of various treatment strategies, including the use of adsorbents, chemical oxidation, and biological degradation. However, the effect of algicidal bacteria on MC production and release has not been fully studied. Despite the potential of algicidal bacteria in bloom management, their impact on MC production and release remains poorly understood, a knowledge gap this study aims to fill. According to previous studies, when the dose of algicidal bacteria exceeds the threshold concentration, the intracellular MCs can be decreased, regardless of the lytic mechanism (Wijesooriya et al., 2021). Our study builds on these findings by investigating the specific thresholds and the underlying mechanisms by which algicidal bacteria reduce intracellular MC concentrations. The reduction in intracellular MC concentration may be influenced by a variety of factors, including but not limited to the downregulation of gene expression (Xuan et al., 2017). For instance, an increase in the release ratio of MCs into the extracellular fraction or the conversion of intracellular forms into by-products through interactions with intracellular molecules may also be equally important processes. However, some studies have shown the opposite results, where the extracellular MC concentration of high-dose algicidal bacteria is higher than that of low-dose bacteria (Li et al., 2021). It has been reported that MC-degrading bacteria and algicidal bacteria have been successfully isolated, which confirms the coexistence of algae-algicide bacteria and MC-degrading bacteria (Morón-López et al., 2023). This coexistence highlights the potential for developing bacterial strains that can both control algal blooms and mitigate the associated toxin risks.

In addition, studies have found that the biodegradation activity of MC-degrading bacteria can be induced under certain conditions, confirming the existence of a single strain with both *Microcystis aeruginosa* (*M. aeruginosa*) and MC-degrading activities (Benegas et al., 2021). However, so far, there have been relatively few studies on the degradation of toxic *M. aeruginosa* and MCs by a single bacterial strain in co-cultured *M. aeruginosa* bacterial systems (Crettaz-minaglia et al., 2020). This study addresses this gap by investigating the potential of a single bacterial strain to degrade both the algal biomass and the toxins, providing a comprehensive approach to cyanobacterial bloom management. By elucidating these mechanisms, our study not only advances the fundamental science of microbial interactions but also paves the way for the development of novel biotechnological solutions for water quality management. In this study, a *Bacillus subtilis* S4 (S4) strain was successfully isolated and screened from the scum of Taihu Lake, which has high algicidal activity against *M. aeruginosa* and strongly reduces the content of MCs. Under the co-cultivation of *M. aeruginosa* and bacteria, the quantitative reactions of algae cells were investigated. The kinetics of MC release and biodegradation were evaluated. The combined activities of the S4 strain and its metabolites have successfully removed *M. aeruginosa* and MCs. The algicidal mode of S4 belonged to an indirect way and the algicidal substances showed stability to protease, acid, base, and heat. Our research provides data support and lays a theoretical foundation for developing an environmentally friendly method to reduce the occurrence of harmful cyanobacterial blooms.

## 2 Materials and methods

### 2.1 Materials

The *M. aeruginosa* FACHB 905 strain was sourced from the Freshwater Algae Culture Collection at the Institute of Hydrobiology, Chinese Academy of Sciences, Wuhan, Hubei, China. Concurrently, the algicidal bacterium was characterized using samples collected from the scum of cyanobacterial blooms in Taihu Lake. Samples were obtained by carefully skimming the lake’s surface with a sterile tool during the peak bloom period, ensuring a representative collection of cyanobacteria-associated bacteria. The collected samples were promptly stored in sterile containers and kept on ice until laboratory analysis. Polymerase chain reaction (PCR) primers were synthesized at Hubei Qingke Biotechnology Co., Ltd. The raw materials of Luria-Bertani (LB) and BG11 medium, used for bacterial and *M. aeruginosa* cultures, respectively, were purchased from China National Pharmaceutical Group Chemical Reagent Co., Ltd., Shanghai, China.

### 2.2 *Microcystis aeruginosa* and bacteria culture

The *M. aeruginosa* FACHB 905 strain was cultured under controlled conditions to simulate its natural environment. The culture conditions included a 12 h (light):12 h (dark) photoperiod, a temperature maintained at 25 ± 1°C, and an illumination condition of 50 μmol photons m^−2^ s^−1^. These conditions were chosen to reflect optimal growth parameters for *M. aeruginosa* as per established protocols (Liu et al., 2018). The pH of the culture medium was rigorously controlled and maintained at 7.0 ± 0.2 throughout the experiment to ensure consistency and to match the neutral pH range preferred by *M. aeruginosa.* The cyanobacteria scum (5 g, wet weight) was mixed with 50 mL of phosphate-buffered saline (PBS) and shaken at 120 rpm for 30 min at 30°C. The samples were serially diluted in sterile water before being inoculated into nutrient agar (NA, 2% agar) plates in 0.1 mL aliquots of each dilution. Approximately 100 mL of LB medium containing isolated bacteria was cultured for 24 h at 30°C and 150 rpm to allow for the bacterial culture’s proliferation. This step was essential to obtain a sufficient quantity of the bacterial strain for subsequent experimental procedures.

### 2.3 Characterization of the algicidal bacterium

By examining the sequencing of the 16S rRNA gene, the isolated strain was recognized. The TIANGEN.DP302-02 kit (Tiangen Biochemical Technology Co., Ltd., Beijing, China) was used to extract DNA from the bacterial samples in accordance with the manufacturer’s instructions, and the 16S rRNA fragments were examined (Wu et al., 2007). In a thermal cycler (Bio-Rad, Hercules, CA, USA), forward 27F (5′-GAGTTTGATCCTGGCTCAG-3′) and reverse 1492R (5′-ACGGCTACCTTGTTACGACTT-3′) were reversed: 95°C for 4 min; 35 cycles at 94°C for 1 min, 56°C for 1 min, 72°C for 2 min; the final step at 72°C for 15 min, at 4°C for 10 min.

### 2.4 Co-incubation experiment

The S4 strain, isolated from the scum of cyanobacteria in Taihu Lake, was used for all experiments involving the algicidal bacteria. To lessen the effect of adding significant amounts of organic nutrients, washed bacterial suspensions were tested in this investigation. The incubation experiment was divided into four parts: the algicidal bacterium was incubated with *M. aeruginosa* (1 × 10^7^ cells/ml) in 100-ml BG11 medium, in which the bacterial density was 1 × 10^6^ cell/ml, 1 × 10^7^ cell/ml and 1 × 10^8^ cell/ml, respectively; another flask was used as control, where *M. aeruginosa* was cultured in a BG11 medium. All experiments were performed in parallel batch culture (100 mL) in 250 mL Erlenmeyer flasks, and the culture conditions are explained in Section 2.2. The control group composed of *M. aeruginosa* received the same volume of BG11 medium and LB. Three replicates were prepared for each test. A small aliquot (5 mL) was removed after filtration, and the chlorophyll a (Chl.a) concentration was measured. It should be emphasized that the concentration of S4 used in this study, as detailed in Sections 2.7 and 2.8, is based on the optimal S4 concentration of 1 × 10^7^ cells/ml, which was determined as discussed in this subsection.

### 2.5 Algicidal effects of bacteria

*Microcystis aeruginosa* cell counts were monitored daily for 7 days using a hemocytometer and a microscope (Carl Zeiss, Jena, Germany). The inhibitory rate was calculated using the following formula: $\mathrm{Inhibitory rate}\;\left(\%\right)=\left(1-T_{t}/C_{t}\right)\times 100\%$ , where T and C are the concentrations of the treatment and control cells, respectively. For the determination of chlorophyll a, we employed a classic method of pigment extraction and spectrophotometry. The specific procedure involved collecting the treated algal cells by centrifugation, followed by pigment extraction using an organic solvent, such as 90% acetone. The concentration of Chl.a was then measured at a specific wavelength using a spectrophotometer. For further investigation, bacteria that showed algicidal activity against *M. aeruginosa* were chosen.

### 2.6 Analysis of MCs

A 30-mL logarithmic phase culture of *M. aeruginosa* FACHB 905 was injected with 5% (v/v) volume fractions of both bacterial cells and cell-free filtrate. The supernatant was obtained by centrifuging at 5,000 *g* at 4°C to precipitate *M. aeruginosa* cells and assay the extracellular MCs. The cells are washed, precipitated twice, frozen and thawed 3 times, and then centrifuged at 12,000 *g* at 4°C to remove cell fragments. The residual supernatant was used to determine the intracellular MCs. The concentration of MCs in the sample was determined in accordance with the Beacon Microcystin Plate Kit’s (Beacon Analytical Systems, Saco, Maine, USA) instructions. Prior to conducting ELISA analyses, the microcystin samples were diluted in a ratio ranging from 1:10 to 1:20. This dilution strategy was essential due to the kit’s limited detection sensitivity, which spans between 0.1 and 2.0 μg/L. To standardize the conditions for ELISA quantification, the pH of the microcystin samples was carefully adjusted to a neutral pH of approximately 7.0. This adjustment was crucial for minimizing pH-related interferences that could potentially skew the results of both direct and indirect ELISA assays during the analysis phase.

### 2.7 Analysis of the mode action of algicidal strain

To ascertain the strain’s algicidal mode, cell-free supernatant (S4 bacterial secretions) and bacterial cells from the S4 strain were separated using filtration and centrifugation, and then treated with *M. aeruginosa* cultures at a ratio of 10% (v/v), respectively. The algicidal strain was exposed to *M. aeruginosa* for 48 h before obtaining the cell-free supernatant. By suspension filtering with 0.22 μm membranes and centrifuging bacterial cells at 1,800 *g* for 5 min at room temperature, cell-free supernatant was obtained.

### 2.8 Stability of cell-free supernatant

To evaluate the stability of the algicidal chemicals secreted by the S4 under different environmental conditions, a series of tests were conducted. The cell-free supernatant containing the S4 secretion was incubated for 2 h at various temperatures (−80°C, −20°C, 0°C, 30°C, 60°C, and 100°C). Additionally, the pH of the supernatant was adjusted to 3, 7, and 10 using NaOH or HCl, and after 2 h, the pH was returned to 7. Finally, 100 mL (10^6^ cells/ml) of pretreated algal culture was inoculated with 2% (v/v) of the supernatant treated under these two methods. The algicidal activity was measured at 12 h to assess the S4 secretions’ tolerance to heat and acid–base conditions. As previously mentioned, the *M. aeruginosa* cell count and algicidal activity were determined. To simulate freeze–thaw cycles, the supernatant was frozen in liquid nitrogen for 5 min and then subjected to three cycles of freezing and thawing in a 65°C water bath. The supernatant was then cooled to room temperature before adding 2% (v/v) of it to a pretreated algal culture containing 100 mL (10^6^ cells/ml). Each experiment was carried out 3 times at 25°C during the light phase of the light cycle. For a measurement of algicidal activity, *M. aeruginosa* cells were counted at 4, 8, and 12 h.

### 2.9 Statistical analyses

Each experiment was run at least 3 times, with the results shown as the average standard deviation of the three repetitions. One-way analysis of variance (ANOVA) (Statistical Package for the Social Sciences [SPSS] 22.0 for Windows) was used to test for significant differences among all the data collected for this study. A *p*-value of 0.05 was used to denote significance.

## 3 Results and discussion

### 3.1 Isolation and identification of the algicidal strain

**Figure 1:**
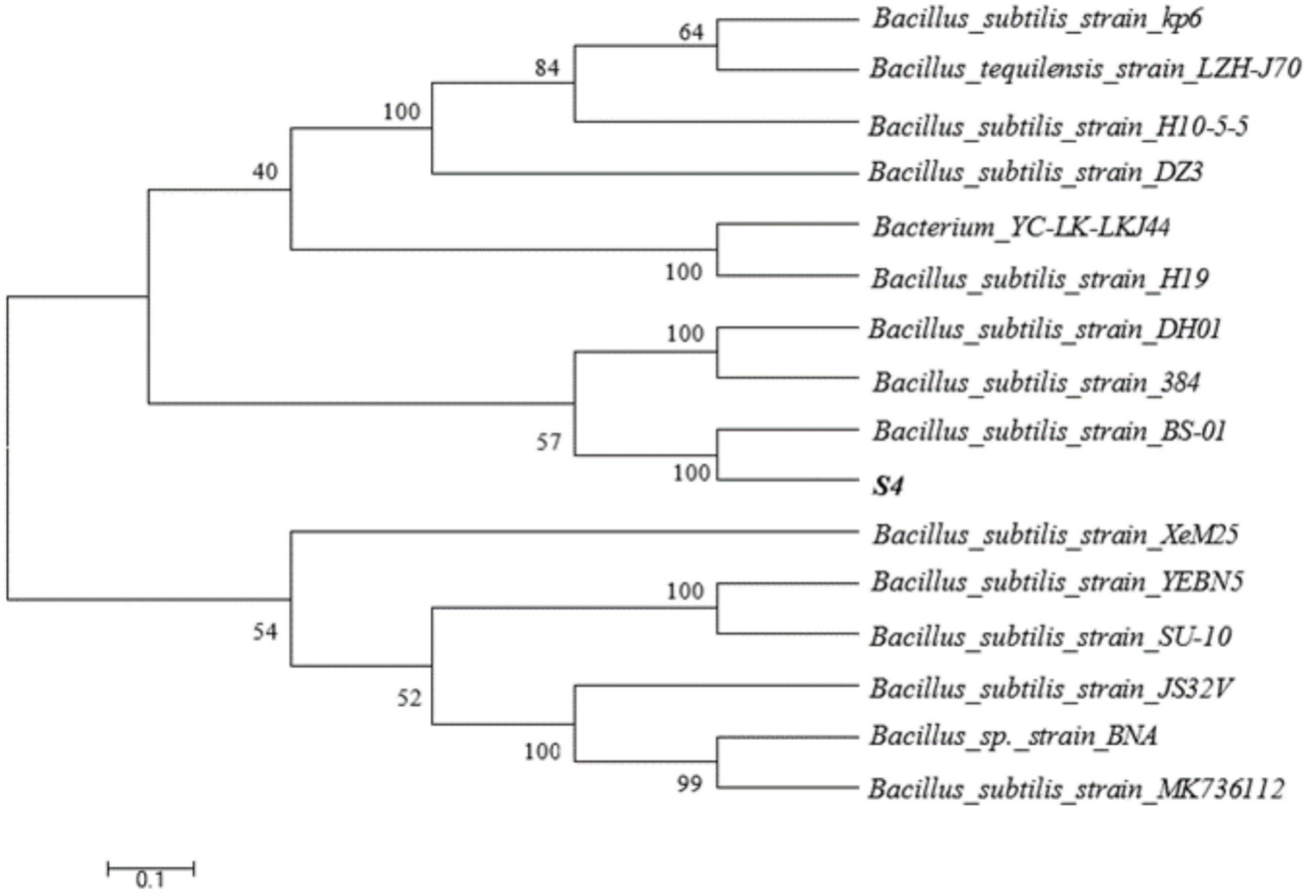
The phylogenetic tree of the algalytic bacterium S4.

The capacity of each of these strains to eradicate *M. aeruginosa* was examined (Supplementary Figure S1). One of these, S4, was chosen for further investigation due to its superior capacity for *M. aeruginosa* degradation, which it can do in just 4 days. From the genomic DNA of the S4, a 1,457 bp fragment of the 16S rRNA gene was amplified (Supplementary Figure S2). The 16S rRNA gene sequence (GenBank accession number: NMDCN00017DJ) was obtained by the GenBank Basic Local Alignment Search Tool (BLAST). The closest *B. subtilis* strains identified using BLAST analysis were *B. subtilis* BS-01 (HM631973). A phylogenetic tree was constructed using MEGA software (Figure 1), indicating that the S4 strain was grouped with *B. subtilis* with high bootstrap values. Many members of *Bacillus* sp. have been reported widely showing algicidal activity, such as *Bacillus* sp. SY-1 (Jeong and Son, 2021), *Bacillus* sp. Lzh-5 (Li et al., 2015), and *Bacillus cereus* N-14 (Nakamura et al., 2003).

### 3.2 Effects of initial bacterial density on algicidal and MCs reduction efficiency

**Figure 2:**
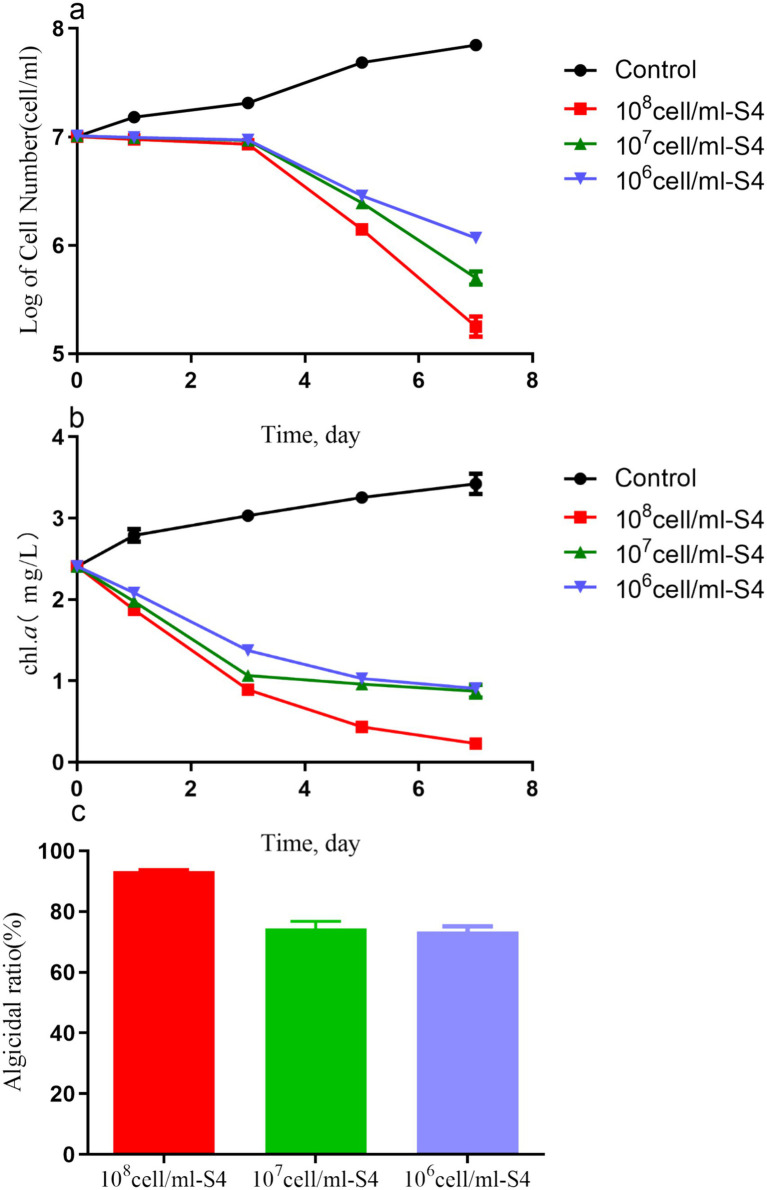
The number of cells (a), Chl.a concentrations (b), and algicidal ratio (c) of *Microcystis aeruginosa* (1 × 10^7^ cell/mL) co-cultured with the S4 strain of different concentrations (10^6^, 10^7^, and 10^8^) for 7 days.

**Figure 3:**
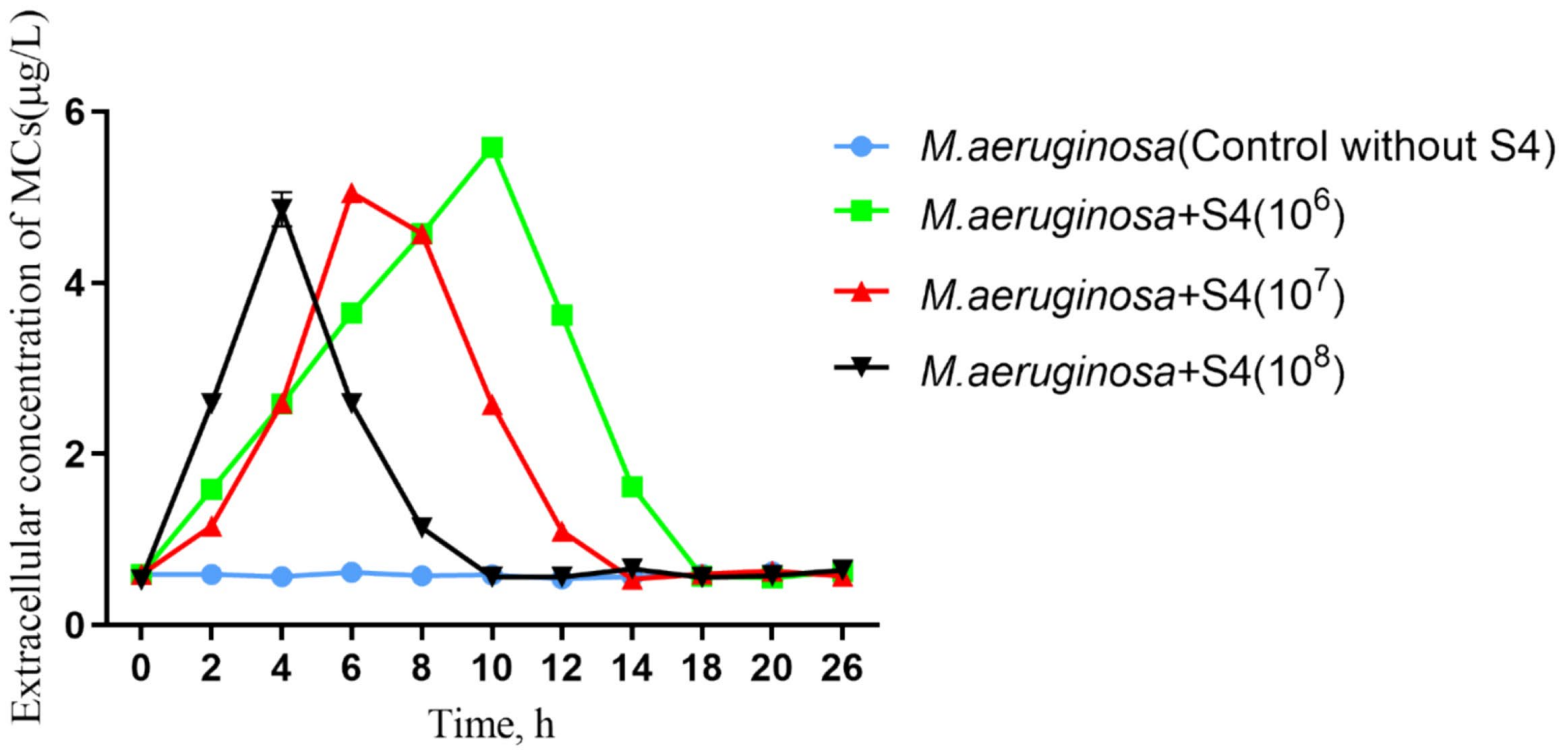
Dynamics of MC concentrations in *Microcystis aeruginosa* and S4 co-cultured systems under varied initial bacterial densities.

Figure 2 illustrates the impact of initial bacterial density on algicidal effectiveness. Significant differences in cell counts were observed in the 10^6^, 10^7^, and 10^8^ cells/ml groups compared to the control group after 7 days of co-cultivation (*p* < 0.05), as shown in Figures 2a,b. At a bacterial density of 10^6^ cells/ml, the algal cell count dropped to 1.27 ± 0.05 × 10^6^ cells/ml, resulting in an algicidal rate of 73.52 ± 2.30%. At bacterial densities of 10^7^ and 10^8^ cells/ml, the algal cell count decreased by orders of magnitude, with corresponding algicidal rates of 93.29 ± 0.46% and 74.54 ± 3.23%, respectively (Figure 2c). Therefore, further research was performed using a bacterial solution of 10^6^ cells/ml concentration, as there was no significant difference among the three concentrations, as shown in Figure 3.

In this study, we found that the initial density of the S4 significantly influences algicidal activity against *M. aeruginosa*, with higher densities leading to greater effects. This aligns with previous studies (Ding et al., 2021; Yang J. et al., 2020; Liu et al., 2022), suggesting that a higher initial bacterial density results in more algicidal compounds being available for direct or indirect lysis of *M. aeruginosa*. The algicidal rate may increase if the bacteria grow to higher densities, highlighting the need to enhance bacterial growth for effective algal bloom management. However, nutrient addition must be carefully balanced to prevent unintended algal growth. Environmental factors such as light, temperature, and pH can modulate bacterial growth and algicidal activity, indicating the need for further research to develop effective and eco-friendly strategies against harmful algal blooms.

The MC concentration in the control group remained stable at approximately 0.59 μg/L, while in co-cultures with S4, MCs peaked at 5.58 μg/L within 10 h before declining (Figure 3), suggesting a biodegradation process. The highest MC concentrations did not differ significantly across different initial S4 densities, indicating a consistent response regardless of density. The timing of MC concentration peaks directly corresponded to the initial S4 density, with the fastest lysis observed at the highest density. The initial increase in MC concentration observed in the co-culture systems (Figure 3) is attributed to the lysis of *M. aeruginosa* cells upon interaction with the S4 strain. This lysis results in the release of preformed MCs, which transiently elevates the extracellular MC levels before the bacteria commence the biodegradation process. The timing of this initial peak in MC concentration corresponds to the initial density of the S4 strain, with higher densities leading to a more rapid release and subsequent degradation of MCs. Our findings support previous research showing enhanced MC degradation at higher bacterial densities (Ndayisenga et al., 2021) and suggest that S4’s algicidal activity contributes to MC degradation, indicating a sustainable process (Yang F. et al., 2020). The kinetics of MC concentration varied with different initial S4 densities, but the overall MC concentrations remained within a narrow range, highlighting the potential of S4 for managing cyanobacterial blooms and MC contamination. Future research should focus on elucidating the mechanisms underlying MC degradation by S4, optimizing conditions for maximum efficacy, and assessing the impact of environmental factors on the strain’s performance to advance the bioremediation of algal blooms.

### 3.3 *Microcystis aeruginosa* lysis and MC degradation patterns

**Figure 4:**
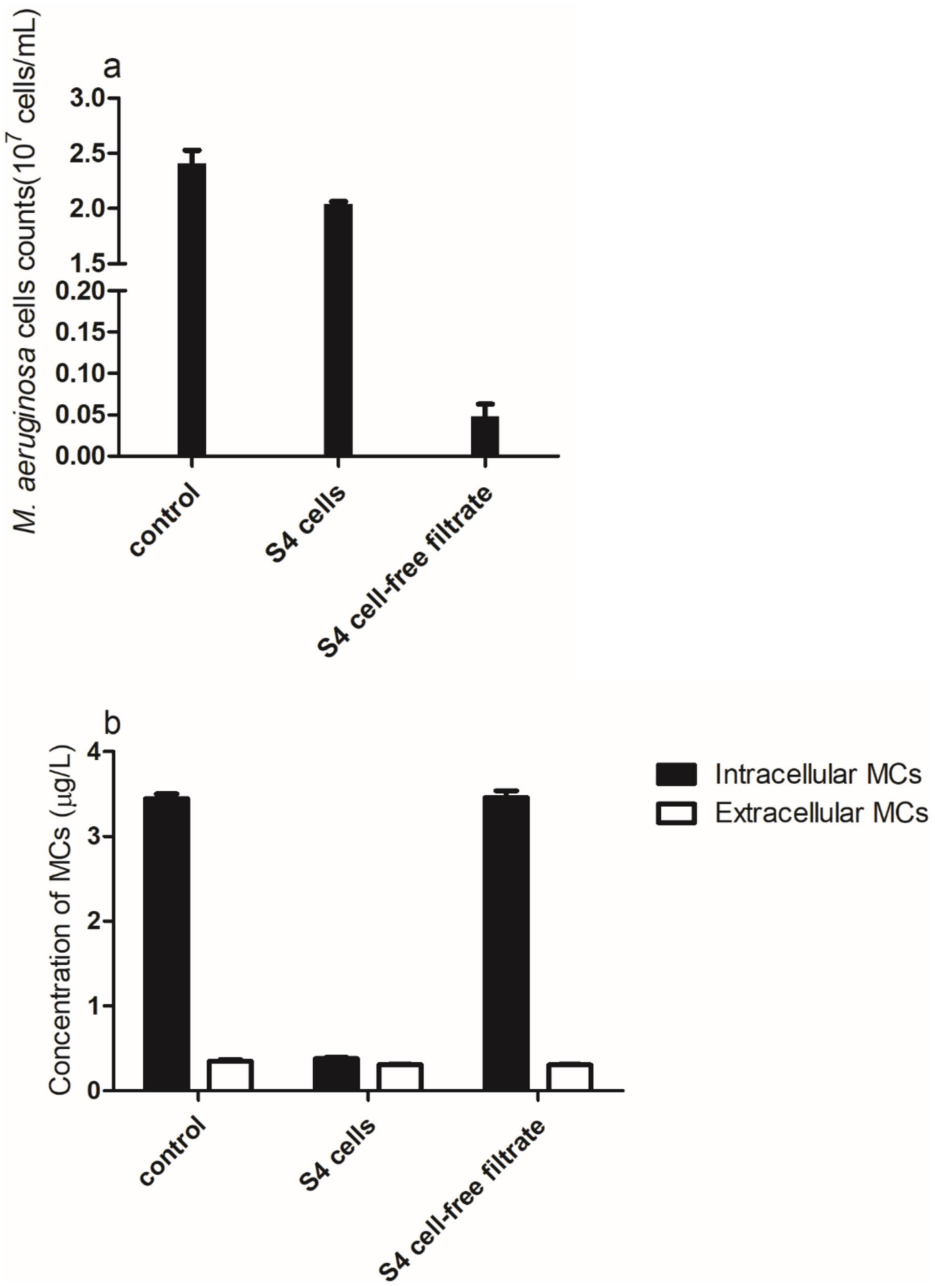
(a) *Microcystis aeruginosa* cell density and (b) concentrations of intra- and extracellular MCs after 24 h in the *M. aeruginosa*/bacterial co-cultured system.

After 24 h of incubation, *M. aeruginosa* and S4 cells showed a modest variation in cell density compared to the control group (Figure 4a). However, the algicidal activity of the S4 cell secretion group was substantially stronger, which is what caused the *M. aeruginosa* cell density to drop by almost 96%. Compared to the control and the group that received additional cell-free filtrate, the extracellular MC concentration reduced almost 2-fold in the bacterial cell incubation group (Figure 4b), and the total MCs (which include both extracellular and intracellular MCs) decreased by approximately 95%. In terms of intracellular MCs, there was no discernible change between the control and the group supplemented with bacterial cell-free filtrate (*p* = 1.73, >0.05), and the degradation efficiency in the bacterial cell-free filtrate group remained at approximately 7%. The degradation efficiency is 89.02% when *M. aeruginosa* and bacterial cells are co-incubated. Earlier studies suggest that 70–90% of planktonic cells lyse algal cells either directly or by creating extracellular chemicals (Effiong et al., 2020). Our findings indicate that the presence of active chemicals in bacterial cell-free filtrate, rather than direct contact between bacterial cells and *M. aeruginosa* cells, is responsible for the algicidal activity. The metabolism of S4 cells, which use MCs as a carbon source for growth, is what leads to MC degradation. In conclusion, *M. aeruginosa* cells and MCs are simultaneously biodegraded due to the combined action of S4 cells and their metabolites.

The biological management of cyanobacterial blooms is a multifaceted approach that includes the removal of cyanobacterial cells and the degradation of their harmful metabolites. Our study supports the view that algicidal compounds are instrumental in the lysis of algal cells, as evidenced by the study of Xu et al. (2022). These compounds, often produced by certain bacterial strains, interfere with the cellular integrity of cyanobacteria such as *M. aeruginosa*, leading to cell rupture and the subsequent release of intracellular contents, including MCs. The biodegradation of MCs, a complex process, is primarily attributed to bacterial enzymatic hydrolysis, as demonstrated by Kumar et al. (2019) and Wei et al. (2023). While our study found only a slight change in MC concentration in the cell secretion group compared to the control group, this does not necessarily mean that bacteria lack the enzymatic machinery to degrade MCs. Instead, it suggests that the degradation process may primarily occur within bacterial cells, which is consistent with the known function of the *mlr* gene cluster. The *mlr* gene cluster, as identified in bacteria capable of degrading MCs, encodes a set of enzymes that work in concert to break down MC molecules (Morón-López et al., 2023). The initial step of MC degradation is often attributed to enzymes encoded by the *mlrA* gene, which cleaves the Adda-Arg bond in the MC molecule (Bourne et al., 1996). Subsequent enzymes, such as those encoded by *mlrB* and *mlrC*, further process the degradation intermediates (Kumar et al., 2019).

Our results show a significant decrease in MC concentration in the presence of live bacterial cells, which points toward an active intracellular degradation process. It is plausible that live bacterial cells internalize and metabolize the released MCs, thereby contributing to their degradation. This process is likely facilitated by the intracellular enzymes of the *mlr* gene cluster, rather than by secreted enzymes. Future studies should focus on characterizing the expression of the mlr gene cluster in bacteria that demonstrate algicidal activity and their ability to degrade MCs. Understanding the regulation and expression of these genes, as well as the specific enzymatic activities involved, will provide insights into the mechanisms by which these bacteria contribute to the biodegradation of MCs.

The bacteria, on the other hand, break down and use the released MCs when living bacterial cells are incubated, as shown by the general fall in the MC levels. The conclusion that algicidal substances cause *M. aeruginosa* cell density to decrease (Figure 4a) and MCs release (Figure 3) in an algae/bacterial co-culture group can, therefore, be reasonably drawn by taking into account the algicidal performance of cell secretion and the MC degradation activity of bacterial cells. As the availability of carbon and nitrogen sources increases, the assimilation of MCs by algal development dominates this process, resulting in a significant decrease in MCs (Figures 3, 4b). As cyanobacteria metabolize MCs for growth, especially under conditions where carbon and nitrogen sources are abundant, the concentration of free MCs in the environment may significantly diminish. This insight could have implications for developing strategies to mitigate the toxicity associated with cyanobacterial blooms. Environmental factors such as nutrient availability, water temperature, and pH can modulate the expression of genes involved in producing algicidal compounds and the enzymatic machinery for MC degradation. Future research should consider these factors to optimize the conditions for effective cyanobacterial management (Song et al., 2024).

### 3.4 Stability of S4 filtrate

**Figure 5:**
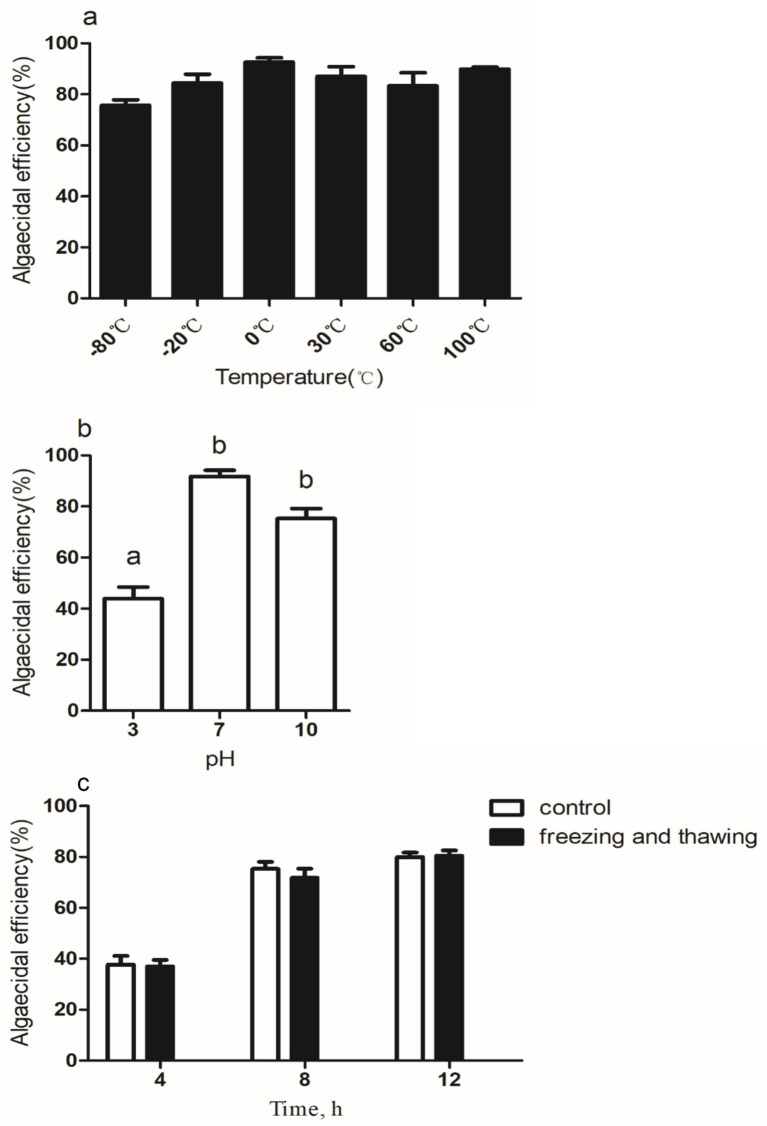
Algicidal effect of strain S4 cell-free supernatant at different temperatures (a), pH (b) conditions, and repeated freeze–thaw cycles (c) treatment *Microcystis aeruginosa* against. Different alphabetical letters, a and b, represent significant differences between groups (*p* < 0.05).

Stability tests showed that the S4 strain’s algicidal chemicals were heat stable, with minor activity changes after exposure to temperatures ranging from −80°C to 100°C (Figure 5a). The pH stability test revealed reduced stability in acidic conditions, particularly at pH 3, but retained activity at pH 7 and 10 (Figure 5b). Freeze–thaw cycles had minimal impact, maintaining algicidal activity even after three cycles (Figure 5c). These results suggest the S4 strain’s potential for aquatic applications. Algicidal bacteria often kill algae directly or indirectly, and the S4 strain likely uses an indirect method, as seen in *Pseudoalteromonas* species (Wang et al., 2020; Zhang et al., 2022). The algicidal chemical’s stability under alkaline conditions, freeze–thaw cycles, and heat resistance indicate it is not a protein and could possibly be a pigment (Wang et al., 2022). Further research is needed to identify the algicidal chemical’s characteristics and subtypes. Our study has certain limitations. The experiments were under controlled conditions, which may not reflect natural aquatic ecosystems’ variability. The tested environmental conditions were limited, and the S4 strain’s response to a broader range of variables, such as salinity and nutrient availability, requires further investigation. Additionally, scaling up from laboratory to field applications may present new challenges, including ecosystem interactions and long-term ecological impacts. Future research should address these limitations by testing a wider range of conditions and exploring long-term field applications.

### 3.5 Innovation of the S4 strain and its potential application in algal bloom management

This study successfully isolated and screened a *B. subtilis* S4 strain with potent algicidal activity against *M. aeruginosa* and the capacity to degrade MCs. Our findings not only substantiate the potential of S4 in controlling harmful algal blooms but also reveal its significant degradation capability for MCs. These discoveries offer new insights in the field of aquatic ecological restoration and bioremediation.

First, the S4 strain demonstrated varying degrees of lytic activity against *M. aeruginosa* and degradation capability for MCs at different densities. As the density of the S4 strain increased from 1 × 10^6^ cells/ml to 1 × 10^8^ cells/ml, the lysis of *M. aeruginosa* and the release of MCs were enhanced; however, MC levels dropped by approximately 90% within 18 h, regardless of bacterial density. This indicates that the combined action of the S4 strain and its metabolites achieved sustainable eradication of both *M. aeruginosa* and MCs, a novel approach not previously reported in the literature (Kong et al., 2022).

Moreover, the algicidal chemicals secreted by S4 exhibited resistance to proteases, acids, bases, and heat, which is unique among the reported algicidal bacteria. This indirect algicidal activity may be mediated by the secretion of stable chemicals that can withstand environmental fluctuations, crucial for persistence and efficacy in practical applications. The stability of these chemicals under various conditions suggests that they are not proteins but possibly pigments or other types of compounds, providing a new direction for further research on the characteristics and subtypes of these chemicals (Ameen et al., 2021).

Our study also found that S4 maintained stable algicidal activity under various environmental conditions. The algicidal chemicals from S4 showed remarkable resilience across a range of temperatures from −80°C to 100°C and retained their activity at pH levels 3–10. This suggests that the algicidal chemicals of S4 are well-suited for application in diverse aquatic environments, which is a significant advantage over other reported strains that may have more limited stability profiles (Azam, 1998).

Although S4 showed significant algicidal and MCs-degradation capabilities under laboratory conditions, we acknowledge the challenges in translating these findings into field applications. Issues such as ecological interactions with other organisms, potential side effects on non-target species, and long-term ecological impacts require further investigation in future studies. The potential application of S4 in natural environments will depend on its ability to persist and maintain activity under variable conditions, which is a critical area for future research (Coyne et al., 2022).

In summary, the discovery of the *B. subtilis* S4 strain provides a novel biotechnological approach to managing harmful algal blooms. Its unique algicidal mechanism and effective degradation of MCs make it a promising biocontrol agent. Future research should focus on optimizing the application conditions for the S4 strain and assessing its long-term effects and ecological impact in natural environments (Crettaz-Minaglia et al., 2020).

## 4 Conclusion

A unique freshwater bacterial strain known as S4 has demonstrated the potential to degradeMCsand exhibit potent algicidal action against *M. aeruginosa*. In the *M. aeruginosa*/bacteria co-culture system, the lysis of *M. aeruginosa* and the release of MCs were accelerated as the density of S4 increased; however, MC levels decreased within 18 h, irrespective of the bacterial density. When comparing the bacterial cell incubation system with the control and the bacterial cell-free filtrate systems, the extracellular and intracellular MCs collectively showed a 95% reduction. The S4, along with its metabolites, has achieved sustainable eradication of both *M. aeruginosa* and MCs.

## Data Availability

The data presented in the study are deposited in the National Microbiology Data Center, accession number: NMDCN00017DJ: https://nmdc.cn/resource/genomics/sequence/detail/NMDCN00017DJ.
