# Supplementary material for: A Bacillus subtilis strain with efficient algaecide of Microcystis aeruginosa and degradation of microcystins
Source: Front Microbiol. 2024 Nov 29;15:1430097. doi: 10.3389/fmicb.2024.1430097 (PMC11638172; doi:10.3389/fmicb.2024.1430097)
Supplement: Supplementary file 1 [file Data_Sheet_1.DOCX]

Figure S1
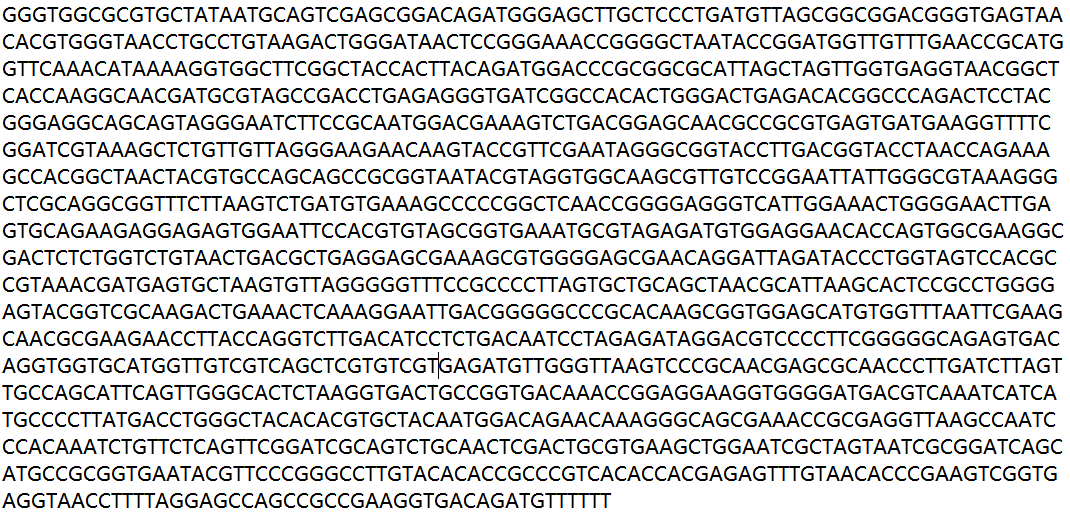

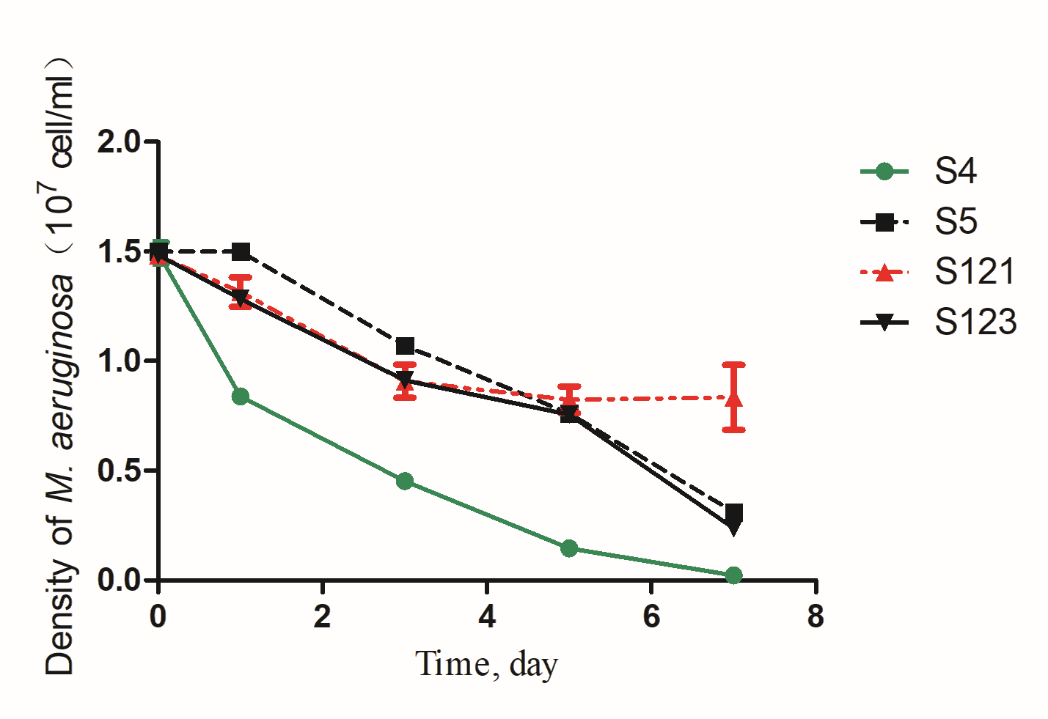
. Algicidal effect of different strains on *Microcystis aeruginosa*

Figure S2. 16S rRNA sequence of strain S4

Table S1 *Bacillus* against cyanobacteria

| **Strain** | **Phylum** | **Class** | **Isolation environment** | **Target cyanobacteria** | **Algicidal mode** | **MCs Degradation** | **Algicidal compounds** | **References** |
| --- | --- | --- | --- | --- | --- | --- | --- | --- |
| *Bacillus subtilis* S4 | Firmicutes | Bacilli | water1 | *Microcystis aeruginosa* | indirect | Highly efficient degradation, 95% Degradation | Algicidal chemicals secreted by S4 that are resistant to proteases, acids, bases, and heat | This study |
| *Bacillus sp.FY-1* | Firmicutes | Bacilli | water1 | *Microcystis aeruginosa* | indirect | unknown | Active substance sensitive to high temperature above 50 ℃ and pH of 5 | (Wu et al. 2024) |
| *Bacillus sp. AK3* | Firmicutes | Bacilli | obtained from Chiang Mai University | *Microcystis sp.* | indirect | Capable, 73% Degradation | Secreted compounds that are resistant to proteases, acids, bases, and heat | (Boonbangkeng et al. 2022) |
| *Bacillus subtilis* | Firmicutes | Bacilli | water1 | colonial cyanobacteria （*Microcystis*） | unknown | unknown | unknown | (Bi et al. 2019) |
| *Bacillus sp. WS8* | Firmicutes | Bacilli | water1 | *Microcystis aeruginosa* | indirect | unknown | unknown | (Donghui Zhang 2019) |
| *Bacillus cereus CZBC1* | Firmicutes | Bacilli | water2 | *Oscillatoria chlorina, Oscillatoria tenuis, Oscillatoria planctonica, Microcystis sp.* | direct and indirect | unknown | unknown | (Hu et al. 2019; Xu et al. 2019) |
| *Bacillus licheniformis Sp34* | Firmicutes | Bacilli | water1 | *Microcystis aeruginosa* | indirect | unknown | unknown | (Jinyu Liu 2019) |
| *Bacillus siamensis Sp37* | Firmicutes | Bacilli | water1 | *Microcystis aeruginosa, Microcystis flos-aquae, Microcystis wesenbergii* | indirect | Capable, Data Unknown | unknown | (Qi Wang et al. 2018) |
| *Bacillus sonorensis Hsn03* | Firmicutes | Bacilli | water2 | *Microcystis aeruginosa* | indirect | unknown | unknown | (Yanting Xu et al. 2018) |
| *Bacillus sp. G6* | Firmicutes | Bacilli | plant | *Microcystis aeruginosa* | indirect | unknown | unknown | (Hongchi Shen et al. 2017) |
| *Bacillus sp. AF-1* | Firmicutes | Bacilli | soil | *Microcystis aeruginosa* | indirect | unknown | unknown | (Huanling Xuan et al. 2017) |
| *Bacillus subtilis* | Firmicutes | Bacilli | unknown | *Microcystis aeruginosa, Chlorella* sp. | indirect | unknown | unknown | (Xin Cheng et al. 2017) |
| *Bacillus mycoides B16* | Firmicutes | Bacilli | water1 | *Microcystis aeruginosa* | indirect | unknown | unknown | (Gumbo et al. 2014; Gumbo et al. 2010; Wang et al. 2017) |
| *Bacillus amyloliquefaciens A10* | Firmicutes | Bacilli | water1 | *Microcystis aeruginosa* | indirect | unknown | unknown | (Zhengyu liu et al. 2016) |
| *Bacillus cereus A21* | Firmicutes | Bacilli | water1 | *Microcystis aeruginosa* | indirect | unknown | unknown | (Zhengyu liu et al. 2016) |
| *Bacillus* sp. *FS1* | Firmicutes | Bacilli | water1 | *Microcystis aeruginosa* | indirect | unknown | unknown | (Mu et al. 2015) |
| *Bacillus sp. Lzh-5* | Firmicutes | Bacilli | water1 | *Microcystis aeruginosa* | indirect | unknown | hexahydropyrrolo[1,2-a]pyrazine-1,4-dione, 3-isopropyl-hexahydropyrrolo[1,2-a]pyrazine-1,4-dione | (Li et al. 2015) |
| *Bacillus sp. SSAL-1* | Firmicutes | Bacilli | laboratory environment | *Anabaen flos-aquae* | indirect | unknown | unknown | (Sun et al. 2015) |
| *Bacillus sp. SSAL-6* | Firmicutes | Bacilli | laboratory environment | *Anabaen flos-aquae* | indirect | unknown | unknown | (Sun et al. 2015) |
| *Bacillus methylotrophicus Ma-B1* | Firmicutes | Bacilli | water1 | *Microcystis aeruginosa* | indirect | unknown | unknown | (Chunmei Zhai et al. 2014) |
| *Bacillus sp. B50* | Firmicutes | Bacilli | water1 | *Microcystis aeruginosa* | indirect | unknown | unknown | (Shao et al. 2014) |
| *Bacillus amyloliquefaciens FZB42* | Firmicutes | Bacilli | soil | *Microcystis aeruginosa, Aphanizomenon flos-aquae, Nostoc* sp.*, Anabaena* sp. | indirect | unknown | bacilysin | (Borriss et al. 2011; Liming Wu et al. 2014) |
| *Bacillus* sp. *T1* | Firmicutes | Bacilli | unknown | *Microcystis aeruginosa* | indirect | unknown | unknown | (Bo XU 2013) |
| *Bacillus* sp. *N25-2* | Firmicutes | Bacilli | activated sludge | *Microcystis aeruginosa* | indirect | unknown | unknown | (Qiu et al. 2011) |
| *Bacillus sp. J1* | Firmicutes | Bacilli | water1 | *Microcystis aeruginosa* | indirect | unknown | unknown | (Li Jin et al. 2010) |
| *Bacillus brevis H1* | Firmicutes | Bacilli | water1 | *Microcystis aeruginosa* | indirect | unknown | unknown | (Huan Hu 2010) |

Water1: waters with cyanobacteria indicated by literatures; water2: not sure if there are cyanobacteria in the water according to literatures.

**References**

1. Wu L, Zhou X, Zhu Y, et al. (2024). Pseudomonas ZY-1 and Bacillus FY-1 protecting the rice seedlings from the harm of Pseudomonas aeruginosa via indirect seawead lysis. BMC microbiology, 24(1): 375. Boonbangkeng D, Thiemsorn W, Ruangrit K, et al. (2022). Promoting the simultaneous removal of Microcystis bloom and microcystin-RR by Bacillus sp. AK3 immobilized on floating porous glass pellets. Journal of Applied Phycology, 34(3): 1513-1525.
2. Bi, X., Dai, W., Wang, X., Dong, S., Zhang, S., Zhang, D., Shi, H. (2019). Effects of Bacillus subtilis on the growth, colony maintenance, and attached bacterial community composition of colonial cyanobacteria. Environ Sci Pollut Res Int, 26, 14977-14987. doi:10.1007/s11356-019-04902-y
3. Donghui Zhang (2019). Isolation, identification, algae-dissolving characteristics and algae dissolving mechanism of a native algicidal bacteria in the Taihu Lake basin. Beijing Jiaotong University
4. Hu, X.J., Xu, Y., Su, H.C., Xu, W.J., Wang, L.H., Xu, Y.N., Li, Z.J., Cao, Y.C., Wen, G.L. (2019). Algicidal bacterium CZBC1 inhibits the growth of Oscillatoria chlorina, Oscillatoria tenuis, and Oscillatoria planctonica. AMB Express, 9, 144. doi:10.1186/s13568-019-0872-8
5. Li, Z., Geng, M., Yang, H. (2015). Algicidal activity of Bacillus sp. Lzh-5 and its algicidal compounds against Microcystis aeruginosa. Appl Microbiol Biotechnol, 99, 981-990. doi:10.1007/s00253-014-6043-6
6. Qi Wang, Simon Paulina, Jinyu Liu, Yuxin Chi, Xianzhu Dai, Hongxia Du, Xiaohui Zhang, Yasuo Igarashi, Caiyun Yan, Feng Luo (2018). Identification of algicidal bacterium Sp37. Microbiology China, 45, 2614−2623.
7. Xu, W., Xu, Y., Huang, X., Hu, X., Xu, Y., Su, H., Li, Z., Yang, K., Wen, G., Cao, Y. (2019). Addition of algicidal bacterium CZBC1 and molasses to inhibit cyanobacteria and improve microbial communities, water quality and shrimp performance in culture systems. Aquaculture, 502, 303-311. doi:10.1016/j.aquaculture.2018.12.063
8. Gumbo, J.R., Cloete, T.E., van Zyl, G.J., Sommerville, J.E. (2014). The viability assessment of Microcystis aeruginosa cells after co-culturing with Bacillus mycoides B16 using flow cytometry. Phys Chem Earth, 72-75, 24-33. doi:10.1016/j.pce.2014.09.004
9. Gumbo, J.R., Ross, G., Cloete, T.E. (2010). The isolation and identification of predatory bacteria from a Microcystis algal bloom. African Journal of Biotechnology, 9, 663-671.
10. Bo XU JY, Ruxian Xiong,Lihong Miao (2013) Research on characteristics of the active substances of alage-lysing production by a Bacillus strain. Journal of Wuhan Polytechnic University 32:28-30+39
11. Borriss R, Chen XH, Rueckert C, Blom J, Becker A, Baumgarth B, Fan B, Pukall R, Schumann P, Sproer C (2011) Relationship of Bacillus amyloliquefaciens clades associated with strains DSM 7T and FZB42T: a proposal for Bacillus amyloliquefaciens subsp. amyloliquefaciens subsp. nov. and Bacillus amyloliquefaciens subsp. plantarum subsp. nov. based on complete genome. International Journal of Systematic & Evolutionary Microbiology 61:1786-1801
12. Chunmei Zhai, Changhong Liu, Lu Lyu (2014) Interaction between Microcystis aeruginosa and Bacterium Ma-B1 Strain within Phycosphere. Research of Environmental Sciences 27: 704-710
13. Hongchi Shen, Ruisong Pan, Xupeng Wu, Qingqing Cai, Wenyi Zhang (2017) Isolation and identification of algicidal bacteria from reed roots in Baidu Port of Taihu Lake and it's lytic effect. Journal of Civil And Environmental Engineering 39:123-128
14. Huan Hu (2010) Studies on lytic characterization of Microcystis aeruginosa by algicidal bacterium H1 and Multiple algicidal microorganisms. Fudan University
15. Huanling Xuan, Da X, Jing Li, Xiaohui Zhang, Caiyun Yang, Luo, F (2017) A Bacillus sp. strain with antagonistic activity against Fusarium graminearum kills Microcystis aeruginosa selectively. Sci Total Environ 583:214-221 doi:10.1016/j.scitotenv.2017.01.055
16. Jinyu Liu (2019) Studies on the algicidal characteristics and mechanisms of Bacillus licheniformis Sp34 and its algicidal substances Southwest University
17. Li Jin, Zhaopu Liu, Gengmao Zhao, Hui Wang, Lei Chen (2010) Effects of an algae-lysing bacterium on the growth of Microcystis aeruginosa and its identification. China Environmental Science 30:222-227
18. Liming Wu, Huijun Wu , Lina Chen, Shanshan Xie , Haoyu Zang, Borriss Rainer, Xuewen Gao (2014) Bacilysin from Bacillus amyloliquefaciens FZB42 has specific bactericidal activity against harmful algal bloom species. Applied and environmental microbiology 80:7512-7520
19. Mu R, Jia J, Zhang S (2015) Initial investigation on algicidal effect and mechanism of algae-lytic bacteria FS1. Journal of Microbiology 35:16-20
20. Qiu X, Qian Y, Zhou R, Zhou Y, Wang Y, Tan Y (2011) Isolation and algicidal effect of algicidal bacterium strain N25. Journal of Shanghai Jiaotong University(Medical Science) 31:1375-1379
21. Shao J, Jiang Y, Wang Z, Peng L, Luo S, Gu J, Li R (2014) Interactions between algicidal bacteria and the cyanobacterium Microcystis aeruginosa: lytic characteristics and physiological responses in the cyanobacteria. International Journal of Environmental Science and Technology 11:469-476 doi:10.1007/s13762-013-0205-4
22. Sun XM, Zheng PZ, Guo LL, Shen JY (2015) Algae-lysing characteristics of two algicidal bacteria.771-774 doi:10.1201/b18135-157
23. Wang J, Hong G, Zhang J (2017) Identification of an algae-lysing bacterium and algicidal mechanism on Microcystis Aeruginosa. Journal of Anhui Jianzhu University 25:19-23
24. Xin Cheng, Kuntai Li, Lin Huang (2017) Research on growth characteristics and algicidal effects of Bacillus subtilis. Biotechnology Bulletin 033:120-125
25. Yanting Xu, Ruixue Song, Congqi Tian, Yi Li (2018) Isolation and identification of Bacillus sp. hsn03 with algicidal activity on Microcystis aeruginosa. Microbiology China 45:58-68
26. Zhengyu liu, Xibing Ning, Wenli Li, Zhijian Wang, Shubin Shan, Xiaohui Li (2016) Isolation and primary study of three algicidal bacteria strains of Tai lake. Food and Fermentation Industries 042:59-64
